# Public views of the uk media and government reaction to the 2009 swine flu pandemic

**DOI:** 10.1186/1471-2458-10-697

**Published:** 2010-11-15

**Authors:** Shona Hilton, Emily Smith

**Affiliations:** 1 MRC Social and Public Health Sciences Unit, Glasgow, UK

## Abstract

**Background:**

The first cases of influenza A/H1N1 (swine flu) were confirmed in the UK on 27th April 2009, after a novel virus first identified in Mexico rapidly evolved into a pandemic. The swine flu outbreak was the first pandemic in more than 40 years and for many, their first encounter with a major influenza outbreak. This study examines public understandings of the pandemic, exploring how people deciphered the threat and perceived they could control the risks.

**Methods:**

Purposive sampling was used to recruit seventy three people (61 women and 12 men) to take part in 14 focus group discussions around the time of the second wave in swine flu cases.

**Results:**

These discussions showed that there was little evidence of the public over-reacting, that people believed the threat of contracting swine flu was inevitable, and that they assessed their own self-efficacy for protecting against it to be low. Respondents assessed a greater risk to their health from the vaccine than from the disease. Such findings could have led to apathy about following the UK Governments recommended health protective behaviours, and a sub-optimal level of vaccine uptake. More generally, people were confused about the difference between seasonal influenza and swine flu and their vaccines.

**Conclusions:**

This research suggests a gap in public understandings which could hinder attempts to communicate about novel flu viruses in the future. There was general support for the government's handling of the pandemic, although its public awareness campaign was deemed ineffectual as few people changed their current hand hygiene practices. There was less support for the media who were deemed to have over-reported the swine flu pandemic.

## Background

On 27th April 2009 cases of influenza A/H1N1 (swine flu) were confirmed in the United Kingdom (UK) after a novel virus first identified in Mexico rapidly evolved into a pandemic [1]. The swine flu outbreak was the first pandemic in more than 40 years [2] and for many, their first encounter with a major influenza outbreak. The World Health Organisation (WHO), sensitised to the possibility of a serious pandemic influenza outbreak following recent outbreaks of Severe Acute Respiratory Syndrome (SARS) and influenza H5N1 (avian flu), had encouraged governments to coordinate a strategic public health response for dealing with such an event [3]. In the spring of 2009, as part of the UK government's pandemic response, a public awareness campaign was launched to slow the potential spread of swine flu by encouraging people to adopt protective health behaviours such as hand washing and using tissues. More broadly, communication efforts centred on explaining to the public the scientific uncertainty which surrounded this novel virus, and scientists, notably those working at the intersections of epidemiology and statistical modelling, monitored and explained scientific predictions of its spread and potential severity. Scientists identified key differences between swine flu and seasonal influenza. Swine flu showed more critical illness among young people [4] (as younger generations were thought to have no natural immunity from previous exposure to a similar influenza A virus) and to be associated with severe viral pneumonitis [5]. Predictions of between 5% and 30% of the UK population contracting the virus and up to 65,000 deaths captured media attention [6].

In the summer of 2009 UK newsprint coverage was at its height, coinciding with the first wave in swine flu cases. (Hilton, in submission) But by autumn 2009, despite a second surge in cases, swine flu was no longer a top news story. In October the swine flu vaccination programme commenced in the UK, first targeting those deemed to be at greatest risk, with the plan of rolling out the programme to the remaining population in the coming months. As the winter progressed it became evident that the virus was not as virulent as first predicted and the stockpile of vaccines would not be necessary, leading to some accusations of "over-hyping the pandemic" [7,8]. By the spring of 2010 mortality data demonstrated that swine flu had been less lethal than first feared and case fatality rates compared favourably with previous influenza pandemics, [9] accounting for less than 500 deaths in the UK [10] and 18,000 worldwide [11]. On the 10^th ^of August 2010, 14 months after WHO declared the pandemic, the Director-General of WHO announced the pandemic officially over.

Unlike other countries, up until this pandemic the UK population had been relatively unaffected by SARS and avian flu, and the government had little experience of planning for a pandemic. In countries affected by those outbreaks research into people's willingness to comply with public health recommendations suggested that this largely depended upon people's understandings and assessment of the perceived risk of contracting the infection and its severity [12-14]. Studies found that people who perceived the greatest threat were most likely to report compliance with government recommendations and that those with least fear were less likely to comply [15-17]. In relation to the swine flu pandemic little empirical evidence has been conducted examining public perceptions. In the UK using aggregate data from 36 national telephone surveys conducted throughout the pandemic, researchers found that public levels of worry about the possibility of catching swine flu were low as were uptake levels for following government recommended hygiene behaviours [18]. Surveys of public opinion on swine flu that have been conducted in other countries have suggested a lack of information [19] and knowledge, [20] and that widespread misconceptions exist [21]. The rapid response and media interest in capturing and reporting swine flu news has meant that the media have been a key source of public health information during the pandemic and questions have been raised about whether the media have contributed to 'over-hyping' the pandemic [22]. However, detailed analysis of newsprint content suggests newspaper coverage was largely proportionate throughout the pandemic [Hilton in submission]. Similarly, the UK Government has also been criticised as having over-reacted to what turned out to be a mild pandemic [7].

This study aims to gain new insights into public understandings of the role of key players in the recent pandemic and to explore how people deciphered the threat and perceived whether they could control the risks. Exploring such perceptions is important in understanding people's willingness to comply with public health recommendations and in order to pinpoint knowledge gaps which may undermine communication efforts.

## Methods

### Sampling

Fourteen focus groups were conducted in Scotland. These groups took place between October 2009 (at the start the second UK peak in swine flu cases) and the end of January 2010 (when swine flu cases had reduced substantially). Purposive sampling was used to recruit participants representing a broad spectrum of opinions and perspectives on swine flu risk, including people in 'at risk' groups as identified by the Department of Health (DoH) http://www.nhs.uk/Conditions/Pandemic-flu/Pages/QA.aspx#mostvulnerable. These groups included people with underlying health problems such as: asthma, chronic heart, kidney or liver disease, chronic neurological diseases, diabetes, and those with suppressed immune systems such as pregnant women.[23] In addition, we recruited people with caring duties, who whilst not at direct risk themselves, had responsibility for caring for more vulnerable persons, such as hospital and community nurses and parents caring for children under the age of five. We also purposively targeted people under the age of 50 years as there was evidence that they may not have acquired immunity from a similar influenza A virus, circulating globally from 1918 to 1957 [24].

### Recruitment

Once target groups were identified, individualised posters, leaflets and adverts were developed to recruit participants. These recruitment materials were placed in a range of community settings including universities, community groups run in leisure/sport and community facilities, and shops. Adverts were run in local newspapers and on websites inviting interested parties to contact the researcher. A small number of participants who fitted our sampling frame were also recruited via snowballing.

### Data collection

Before commencing the group discussion, participants completed a short demographic questionnaire recording information on age, whether they had children, any health issues, and whether they thought that they had had swine flu, and if offered, would accept or reject swine flu vaccination. A topic guide for the discussions was developed from a rigorous review of the literature. This guide was used to explore the following themes: understandings of pandemics; perceptions of swine flu and risk factors; attitudes towards swine flu vaccination and from where people obtained information about swine flu. The group discussions were facilitated by ES and lasted between one and two hours. In order to allow each participant enough time to express their views and share their opinions focus group numbers were kept small with between four and six people in each group. All the groups were audio recorded and transcribed verbatim.

### Analysis

To enable systematic comparisons to be made across the large amounts of data, each transcript was checked and imported into NVivo 7. Data were thematically coded and, following the principle of the constant comparative method and rigorous analysis, [25] each transcript was repeatedly re-examined and cross-compared to identify common themes and underlying reasoning. Once all the relevant extracts of data had been retrieved and checked a coding frame was developed around which to examine the public's perceptions and understandings of swine flu threat and acceptability of the vaccine. Throughout the analysis particular attention was paid to deviant or contradictory cases [26] and to group dynamics, using field note observations [27].

### Reporting data

To report the data we have used concise quotes attributable to an individual where that individual's view was 'typical' of a broader range of views or where the quote was 'atypical', deviating from the common or collective viewpoint. We have also selected group quotes to convey a sense of the dynamic and rich data yielded from these group interactions whilst being mindful from field-notes of any group effect and the fact that all conversation is influenced by the context in which it is generated [28]. For transparency we have identified the question the facilitator asked and have indicated where topics emerged spontaneously from the participants themselves. One of the advantages of using focus group methods is that it can generate rich and dynamic data by encouraging discussion between group members [29]. However sometimes the chaotic nature of focus group conversation can make it difficult to identify all the individuals and this was particularly challenging in this study. As far as possible we have attempted to identify participants, using pseudonyms.

Ethical approval for the study was obtained from the research ethics committee of the University of Glasgow's Law, Business and Social Sciences Faculty.

## Results

Seventy three people (61 women and 12 men) took part in 14 focus group discussions (see table 1). Participants were aged between 16 years of age and 60. Twenty five were parents, of whom more than half had children under the age of five. Seven participants considered that they had had swine flu and thirty six believed that they would accept swine flu vaccination either for themselves or their children if offered. Our purposive sampling strategy meant the sample had nineteen people who deemed themselves at greater risk because of underlying health problems, including asthma (n = 7) and a chronic kidney problem (n = 1), or because they were pregnant (n = 11).

**Table 1:** Focus group participants

Group	Pseudonym (age)	Had SF	Children	Accept vaccine	Greater risk from SF	How concerned about SF infection	How concerned SF vaccine
**FG1**	Rob (35)	N	N	N	N	Concerned	Very concerned
	
	Beth (17)	N	N	N	N	Not at all concerned	Not at all concerned
	
	Dave (18)	Y	N	Y	N	Slightly concerned	Slightly concerned
	
	Ellie (18)	Y	N	N	N	Not at all concerned	Not at all concerned
	
	Sophie (20)	N	N	N	N	Slightly concerned	Slightly concerned

**FG2**	Lorna (19)	N	N	N	N	Not at all concerned	Not at all concerned
	
	Kelly (18)	N	N	N	N	Slightly concerned	Slightly concerned
	
	Rebecca (18)	Y	N	Y	N	Slightly concerned	Not at all concerned
	
	Holly (18)	Y	N	N	N	Not at all concerned	Not at all concerned
	
	Isla (18)	N	N	N	N	Not at all concerned	Not at all concerned
	
	Alison (17)	Y	N	N	N	Slightly concerned	Slightly concerned

**FG3**	Fiona (22)	N	N	Y	N	Slightly concerned	Not at all concerned
	
	Alice (18)	N	N	Y	N	Slightly concerned	Not at all concerned
	
	Karen (24)	N	N	Y	N	Slightly concerned	Not at all concerned
	
	Joanne (19)	N	N	N	N	Not at all concerned	Slightly concerned
	
	Iona (18)	N	N	N	Y Asthma	Missing	Missing

**FG4**	Emma (21)	N	N	Y	N	Slightly concerned	Slightly concerned
	
	Olivia (23)	N	N	Y	N	Slightly concerned	Not at all concerned
	
	Hannah (21)	N	N	Y	N	Slightly concerned	Not at all concerned
	
	Sue (23)	N	N	Y	N	Not at all concerned	Not at all concerned
	
	Eva (22)	N	N	Y	N	Slightly concerned	Not at all concerned
	
	Sarah (22)	N	N	Y	N	Missing	Missing

**FG5**	Mia (17)	N	N	N	N	Slightly concerned	Concerned
	
	George (25)	N	N	Y	Y Asthma	Concerned	Slightly concerned
	
	Julia (20)	N	N	N	N	Slightly concerned	Not at all concerned
	
	Katy (22)	N	N	Y	N	Not at all concerned	Not at all concerned
	
	Lily (26)	N	N	N	N	Slightly concerned	Slightly concerned

**FG6**	Emily (30)	N	N	N	Y Pregnant	Concerned	Concerned
	
	Jenny (31)	N	N	Y	Y Pregnant	Concerned	Slightly concerned
	
	Nicole (33)	N	N	Y	Y Pregnant	Slightly concerned	Slightly concerned
	
	Maria (39)	N	Y	Y	Y Pregnant	Concerned	Slightly concerned
	
	Amy (33)	N	N	N	Y Pregnant	Slightly concerned	Slightly concerned

**FG7**	Erica (39)	N	Y	Y	N	Slightly concerned	Concerned
	
	Mary (41)	N	Y	Missing	N	Not at all concerned	Slightly concerned
	
	Carly (36)	N	Y	Y	N	Slightly concerned	Slightly concerned
	
	Amanda (33)	N	Y	Y	Y Asthma	Concerned	Not at all concerned
	
	Melanie (36)	N	Y	N	N	Slightly concerned	Concerned
	
	Claire (42)	N	Y	N	N	Not at all concerned	Concerned

**FG8**	Evelyn (60)	N	Y	Y	N	Not at all concerned	Not at all concerned
	
	Diane (32)	N	Y	Y	Y Renal problems	Slightly concerned	Slightly concerned
	
	Amber (32)	N	Y	Y	Y Pregnant	Concerned	Slightly concerned
	
	Angela (40)	Y	Y	N	N	Slightly concerned	Not at all concerned
	
	Jackie (35)	N	Y	N	Y Asthma	Slightly concerned	Slightly concerned

**FG9**	Natalie (30)	N	Y	Y	Y Pregnant	Concerned	Concerned
	
	Fran (30)	N	Y	N	Y Pregnant	Slightly concerned	Slightly concerned
	
	Susan (34)	N	Y	Y	N	Slightly concerned	Slightly concerned
	
	Lynda (36)	N	y	Y	N	Concerned	Concerned

**FG10**	Ashlyn (16)	N	N	Y	N	Concerned	Not at all concerned
	
	Steven (28)	N	Y	N	N	Not at all concerned	Not at all concerned
	
	Drew (25)	N	Y	N	Y Asthma	Slightly concerned	Concerned
	
	Layla (28)	N	Y	N	N	Missing	Missing
	
	Erin (27)	N	Y	Y	Y Pregnant Asthma	Very concerned	Concerned
	
	Anna (23)	N	Y	Y	Y Pregnant	Slightly concerned	Not at all concerned

**FG11**	Ruby (19)	N	N	Y	N	Slightly concerned	Not at all concerned
	
	Ciara (20)	N	N	N	N	Slightly concerned	Slightly concerned
	
	Zoe (20)	N	N	Y	Y Asthma	Slightly concerned	Slightly concerned
	
	Lynn (20)	N	N	Y	N	Not at all concerned	Slightly concerned
	
	Valerie (19)	N	N	N	N	Slightly concerned	Not at all concerned

**FG12**	Peter (31)	N	Y	N	N	Not at all concerned	Slightly concerned
	
	Kelly (40)	N	Y	N	Y Asthma	Not at all concerned	Concerned
	
	Lucy (-)	N	Y	Y	Y Pregnant	Concerned	Slightly concerned
	
	Jasmin (32)	N	Y	Y	N	Slightly concerned	Concerned

**FG13**	Kyla (23)	N	N	N	N	Concerned	Concerned
	
	Leah (22)	N	N	N	N	Slightly concerned	Not at all concerned
	
	Alexa (22)	N	N	N	N	Not at all concerned	Slightly concerned
	
	Craig (22)	N	N	Y	N	Slightly concerned	Not at all concerned
	
	Zoe (22)	N	N	Y	N	Not at all concerned	Slightly concerned
	
	Kylie (23)	N	N	N	N	Not at all concerned	Not at all concerned

**FG14**	Cameron (20)	Y	N	Y	N	Not at all concerned	Not at all concerned
	
	Nick (21)	N	N	N	N	Slightly concerned	Slightly concerned
	
	Alan (23)	N	N	N	N	Slightly concerned	Not at all concerned
	
	Stuart (21)	N	N	N	N	Missing	Missing
	
	Jim (22)	N	N	N	N	Not at all concerned	Not at all concerned

### Deciphering the threat

Participants were asked to describe their images of swine flu. Typically, participants reported images of 'Mexico', 'pigs', the man who was depicted sneezing in the DoH swine flu public awareness campaign and people wearing 'face masks'. Some participants mentioned more dramatic images including: 'chaos', 'death', 'borders and airports closing' and 'people being quarantined'. Many of these images appeared to have come directly from the media and it was common for participants to state that they had seen these images on the television, or in newspapers. For example, one participant responded: "The picture I have is from the newspaper, with all the people who were wearing the masks" (Dave, FG1). Another participant recalled: "I just remember it being on TV...all you saw was everyone running about with white masks on so that's kind of the image I have of something very contagious that they can't confine" (Lucy, FG 12). There was agreement across the groups that a pandemic in this century would be unlike past pandemics. As one women considered: "It won't be like ones in the past, years ago. There's so much medical research been done. And things are cleaner, better housing, people are cleaner and the streets are cleaner so a pandemic wouldn't be so bad now" (Lily, FG5).

When asked to discuss how they viewed the threat to the general British population from swine flu, participants across all the groups spoke about how they felt the threat had changed over the months since it first emerged in Mexico. Participants commonly mentioned that before swine flu was confirmed in the UK they assessed it might be: "like another SARS experiences, not coming to much" (Emma, FG4). However, once cases were confirmed in the UK and public health officials appeared unable to contain its spread they viewed it as more threatening. As one woman recalled: "...it was scary when they said they were trying to contain it and then they discovered they couldn't..." (Fran, FG 9). However, these initial fears quickly subsided over the subsequent weeks. The main reason given by participants for this reappraisal of risk was that during the summer wave of swine flu cases, people in Britain had become more familiar with swine flu and less fearful of it through direct experience or knowing someone who had contracted it. A typical view was that: "At first, it was like we're all going to die...but before long we knew we weren't and it was just no big deal" (Angela, FG8). In another group they discussed this issue and considered:

**Beth**: It doesn't really bother me anymore like, when it first came over, it was a big deal, like, 'oooh how many might die,' but now so many people have had it it's not really that big a deal, it's just like, 'oh, it's just the flu'

**Dave**: Nobody's really actually bothered anymore...

**Ellie**: My friends were so un-worried about it. This summer, they were like, 'well, cheap tickets to Cancun'. They all went to Cancun for their like holiday - and they were like 'it's costing us like nothing'

**Sophie**: Yeah, there's no sense of panic. (FG 1)

It was common for participants to view swine flu as more contagious than normal flu, and several participants referred to it as like a dose of "man flu" (Zoe, FG 11; Olivia, FG 4; Dave, FG 1) which participants described as, "like a worse, more severe flu" (Rob, FG 1). Although seven participants believed they had had swine flu, no participants had any friends or family who had died from swine flu and one man summed up the risk as: "You've got more chance of winning the lottery than dying from swine flu... thankfully" (Steven, FG10).

When participants were asked to discuss how they viewed their personal threat from swine flu, it was evident that the threat was weighed up within the context of their assessment of their own health. Participants who deemed themselves to be healthy assessed swine flu as less of a threat. In contrast, those participants with underlying health problems or who considered themselves to be in an 'at risk' group felt most threatened by swine flu. For instance, one women said: "The reason I don't think it'll be bad for me is coz I reckon I've got quite a good immune system so the risk is not there for me" (Layla, FG10). Similarly, in another group participants considered:

**Emma**: I'm personally not worried. I think my immune system is working well and I'm not in the situation of having an illness.

**Sue**: Same. I know like thousands of people have got it, but...I don't personally feel at risk.

**Emma**: Yeah, I'm not really worried about getting it, like I'm not going to die or anything if I get it. Just be like- well obviously it's just flu, so obviously it'd be rubbish, you'd be ill for two weeks... (FG 4)

In the groups with participants who did not view their health as being at risk from swine flu there were many instances of them making light of the pandemic. One participant commented: "There is so much joking about it, like I was on the train and this guy just coughed, and he was like, 'it's ok, it's not mutated, I don't have swine flu' and everyone just laughed. No, I don't think people are worried about it" (Holly, FG 2). Nevertheless, participants were also sensitive towards and displayed concern for people they deemed to be at risk. Participants cited people with chronic health problems such as asthma and those with a vulnerable immune system such as pregnant women and young children to be at greatest risk. Mothers who were caring for young children with health problems seemed especially anxious. In one group a mother explained: "...my son's got quite severe asthma so the whole swine flu thing still panics me quite a bit" (Carly, FG 7). Similarly, pregnant women also displayed greater levels of concern. As one woman explained: "...when I learned about your immunity when you're pregnant - how it is lowered, I've been more worried" (Jenny, FG 6).

### Information sources to assess the threat

Participants identified two key sources as providing information to help them assess the threat from swine flu: the media and the government. Less common were family, friends and health professionals. As one participant said: "I don't think I've picked up anything from anywhere, apart from the media, that's been my only source of input about swine flu" (Jackie, FG 8). It was common for participants to mention that in the early phase of the pandemic they believed that there was too much news coverage and this caused them to feel anxious. For instance Rob remarked: "I listen to Radio One every single morning. Every single morning it would just be like, 'swine flu,' and I was just like, 'shut up, I've had enough'. They keep on so you get the message it's important" (Rob, FG 1). Participants also judged that since the summer of 2009 there had been a decline in media interest. For example, one participant commented: "I've really noticed that the newspapers and stuff aren't paying as much attention to it anymore" (Ciara, FG 11). The rationale participants gave for the decline in coverage was that early predictions had over-estimated the prevalence and severity of swine flu: "It's not as bad as we thought so it isn't going to make the front page of the tabloids" (Peter, FG 12). The media coverage of swine flu was also described by many of the participants as 'scaremongering'. They often expressed concern with how the media deliberately tried to induce unnecessary panic. In one group they concluded:

**Emma**: they've [the media] set about and managed to get everyone, or the majority of people into quite a panic about the whole thing.

**Sarah**: They do on purpose whip up panic and anxiety in people

(FG 4).

Similarly, participants in another group assessed the media's role as an information source, and stated:

**Ellie**: At first they were basically saying 'that we're all doomed.' That's the impression they were giving, and I just found that was very unnecessary.

**Sophie**: Yes the intent to cause hysteria in people. I don't find it myself, but I've seen other people lapping it up and worrying (FG 1).

In three of the groups the media were also criticized for covering swine flu at the expense of more serious diseases which pose a greater threat to lives. For instance, one participant in a group of student nurses commented:

**Mia**: Before swine flu came along, MRSA and C-Diff was in the news like an awful lot, and nowadays you hear like nothing about it, and it is still around - and people like still do need to know about how that can like affect you as well.

**Katy**: No, it shouldn't just be dropped just because something new and big has come along... (FG 5).

Despite using the media as a key information source it was also common for participants to express distrust in the information provided by the media and the motives behind media coverage. One participant explained her frustration and questioned: "One paper tells you one thing, another paper tells you a different thing altogether. So can you actually rely on the media to give you the proper information?" (Amber, FG 8).

The second key source for assessing the threat from swine flu was the government. All of the participants had seen the government's public awareness campaign, 'Catch it, bin it, kill it'. However, almost all described the information contained within the campaign as 'common sense' and 'obvious' and there was a mixed response about its value. Only 8 participants believed that they had increased hand washing or usage of tissues since the campaign. Nevertheless, the government were generally viewed as having handled the swine flu situation reasonably well. A typical view was expressed in Focus Group 13:

**Craig**: They did what I would expect them to have done. I don't think they achieved anything spectacularly impressive, but at the same time, I know they reacted with what they had, and how people expected them to react, so you can't criticise them for having done that.

**Alexa**: Em exactly, they reacted in proportion to the people's expectations.

**Kyla**: And to the perceived threat at the time.

**Craig**: Yeah exactly. (FG 13)

At the time these focus groups took place participants were aware that the UK Government had ordered enough vaccine for the entire population and had begun stock piling the vaccine for the most vulnerable people. Participants commonly expressed the view that they felt reassured by the government having ordered enough vaccines: "I think it's good to have a vaccine, so vulnerable people can feel protected, we're lucky cos other countries have not got in fast enough to get enough [vaccine] for their whole population" (Diane, FG 8).

### Controlling the risks

Participants across the groups seemed well informed about how swine flu could be transmitted from person to person, through sneezing and touching contaminated objects, and knew of the recommended infection control measures they should adopt. Participants commonly reported knowing that government recommendations were hand washing and using tissues when sneezing. However, most participants did not believe they had changed their current hand washing practices or incorporated any new practices into their daily routine. For instance, Fran (FG 9) stated: "I can't say I've changed anything cos I already do my hand washing, so others might benefit from this advice more really". In this respect it was common for participants to view the government campaign as being of potential benefit for others. A few participants did mention avoiding public transport and wearing gloves when touching communal objects such as hand rails, ticket machines and toilet door handles (FG5, FG6, FG 8, FG 12). No participants spoke of using facial masks.

Participants were asked about their views on the development of a swine flu vaccine. The issue of vaccine safety cropped up in all the groups. The key concerns expressed were about the speed of its development and whether it had been sufficiently trialled. For instance, one participant commented: "... there's not enough trials being done before it's being sent out because they're in such a rush to get it out to people" (Kelly, FG 2). It was also common for participants to link perceptions about rushed trials to concerns about long-term side-effects. For example, one participant said: "they haven't tested the implications on your body in five years time or ten years time, which is what worries me" (Mia, FG 5). Although all the groups discussed concerns about vaccine safety and questioned whether enough research had been done to assure its use, one participant offered an alternative assessment:

It's kind of they're damned if they do, and damned if they don't. I think if they didn't come up with a vaccine for swine flu, we'd all be like, 'well what are they playing at? Come on - get it sorted.' And now they have, it's like, 'oh, that was a bit fast, that was too fast for my liking' (Sophie, FG 1).

When asked about the differences between the vaccines for swine flu and for seasonal flu, participants were typically unsure. For example, one participant asked: "This is a really stupid question, but if normal flu is the same, is H1N1, and swine flu is H1N1, what's the difference between the vaccines?" (Beth, FG 1). Participants also knew little about seasonal influenza vaccines and wondered whether they would be effective against swine flu. This confusion led three participants to suggest that the seasonal flu vaccine must be a safer option because it had been effective for years (Dave, FG 1; Anna, FG 10; Zoe, FG 13).

When participants were asked whether they would accept or reject swine flu vaccination if offered, they were split between accepting, refusing vaccination and being undecided. Many of the participants discussed weighing up the risks and benefits of vaccination as a way of making the decision. One man reasoned:

None of us have got underlying health issues, none of us get ill very often so the risks from swine flu seems to be quite slim and so we don't need it (vaccine) actually, so that's in my calculations anyway (Peter, FG 12).

Whilst for many participants this was a hypothetical question, the pregnant women in the group discussions had all been offered the vaccine and seemed anxious about uncritically accepting it. It was typical for these participants to express concern over the safety of the vaccine. For instance, one woman explained: "I think what worries me is the testing. Has it been tested and how do we know, by taking it, that it's not going to affect our baby so I won't take it" (Emily, FG 6). Three other participants also stated they were not planning to have the vaccine and mentioned the case of Thalidomide as highlighting the dangers of insufficiently tested drugs taken during pregnancy. However, one participant was scathing of links between the swine flu vaccination and Thalidomide, stating:

I think that Thalidomide and things like that, that's completely unrelated. That's got absolutely nothing to do with it, so to me, that's borne of ignorance [...] I have concerns, but not concerns enough not to get the vaccination. (Nicole, FG 6).

It was common for these women to mention that they felt there were mixed messages regarding medication and pregnancy. For example one woman commented: "it concerns me cos you're so drummed in when you're pregnant about not taking any medication" (Amy, FG 6). Trust was an issue that emerged in several group discussions, particularly when pregnant women were discussing their decisions about whether to have the vaccine. Many participants were unsure whether they could trust the advice to vaccinate.

The other groups of participants that expressed concerns about vaccine safety were mothers with young children. One mother questioned: "do they know how much of the vaccine to put into his body? It's not been researched for that long. I would rather just wait, he's healthy, wait and see and fingers crossed, he wouldn't ever you know, contract swine flu" (Jasmin, FG 12). Similarly, another mother weighed up the risks and considered: "I wouldn't do it [vaccinate] because I don't think the risk of swine flu is great enough for my children" (Mary, FG 7). However, there were participants who were keen to have their children vaccinated, particularly if they felt their child was vulnerable to infection. For example, one mother whose son had asthma believed her son was: "quite prone to illnesses and chest infections" (Kelly, FG12) so reasoned that he would benefit from additional protection. There was also one mother whose child did not have any underlying health issues but was keen to have her son vaccinated. She commented: "...younger kids tend to get more illnesses because they play together you know and everything. So I think their age group is a vital group to give it to" (Amber, FG 8). A few participants also spoke about accepting the vaccine altruistically as a means of protecting vulnerable populations. For instance, one participant stated: "it's about protecting the vulnerable people... it's more protection you know of vulnerable people at the end of the day than anything else" (Emily, FG6).

## Discussion

The aim of this study was to conduct an in-depth exploration of public perceptions and concerns during the swine flu pandemic in order to gain insights into how people deciphered the threat and perceived they could control the risks, in order to pinpoint knowledge gaps and shape future pandemic communication. Perhaps one limitation could be that there were more women than men who took part in this study (almost a ratio of 5:1). However, we were particularly interested in hearing the views from people in 'at risk' groups which included pregnant women and from those who have caring duties such as nurses and mothers of young children, therefore we purposively sought their views to fully explore various issues.

In the early months after swine flu first emerged, and whilst there remained scientific uncertainty about its level of threat, concerns appeared to be at their height. These concerns reduced as people became more familiar with swine flu. Most of these focus groups were conducted around the time of the second wave in UK swine flu cases in the autumn of 2009. Consistent with a UK survey [18] and in contrast to public attitude studies conducted in other countries [19,21] there was evidence that participants in this study perceived that there was an inevitability to contracting swine flu and assessed self-efficacy for protecting oneself as low, based on the belief that swine flu was more contagious than normal seasonal influenza. This may have resulted from some confusion around the phrase 'containment phase'. On the 2^nd ^of July 2009 the DoH moved from containment (outbreak management) to the treatment phase [30]. The public may have understood this move from containment as a failure to stop it completely as implied by several participants and findings from an independent review of the UK Government handling of swine flu http://www.cabinetoffice.gov.uk/media/416533/the2009influenzapandemic-review.pdf. Despite believing there was a high likelihood of contracting swine flu, there was little evidence that participants assessed swine flu as life threatening for healthy people. This finding adds further weight to the notion that even during the period when there were more swine flu cases, the public were not unduly anxious [22]. It is known that risk perceptions about the seriousness of a disease and perceived personal vulnerability are key factors in whether people take action to protect health [31]. Evidence from this study confirms this. Participants who assessed that they were in good health did not perceive swine flu as a threat to their health, while those in contact with more vulnerable groups, or who assessed their own health as compromised deemed themselves at greater risk from harm from swine flu. These findings resonate with large population opinion surveys [22,21] and with evidence on disease perceptions [32] and factors in vaccine decision-making [33].

In relation to controlling the risk through swine flu vaccination, our findings are comparable with those of a study in which health workers' concerns about vaccine safety and efficacy were key reasons for swine flu vaccine refusal [34]. Concerns about vaccine safety were raised in all the groups relating to the speed of vaccine development and whether or not it had been sufficiently trialled. Participants did not debate whether natural immunity would be better than artificial immunity, or appear to be aware that this is the first time a vaccine had been developed in rapid response to a pandemic, or that each year seasonal flu vaccines are developed in response to viral changes. Indeed, people were unsure and confused about the difference between seasonal flu vaccines and the swine flu vaccine. This suggests that had the vaccination programme been rolled out to the general population, the actual uptake would have depended on people's assessment of the risks associated with the vaccine and swine flu. Without the assurances of clinical evidence on efficacy and safety of the vaccine there may have been sub-optimal uptake. Confusion between the different types of influenza vaccines suggests a need to clarify misconceptions and to explain to the public the scientific evidence behind influenza vaccine programmes. Further, our findings underline the importance of providing timely, targeted information when vulnerable groups in the population are identified for immunisation.

Participants were well informed about the government's public awareness campaign, 'Catch it, bin it, kill it', and about how swine flu could be transmitted from person to person. However, few indicated that they had changed their behaviours, a finding consistent with a large survey examining behaviours during the early phase of swine flu pandemic [22]. The reason given for this was that they believed that they already practiced 'good' hand hygiene and considered the campaign was directed at others. Another reason described in other research was that high compliance with health related recommendations is more likely to occur among people who deem themselves at greatest risk [35]. Similar to findings from other countries with more experience of influenza pandemic preparedness [21,19], there was general support for the government's handling of the pandemic. This research was largely conducted before concerns were expressed in the media about the government spending money on over-ordering vaccines subsequently deemed unnecessary. Participants in this study viewed the stockpiling of vaccines as a reassuring sign that the government were taking the risk seriously. At the time of the focus groups, this action was viewed as a proportionate response and seemed to contribute towards building public confidence that the government was in control and an authoritative figure in protecting its population, even if claims about the mass vaccination programme were hastily developed on some false scientific assumptions [36].

The mass media were identified as a primary source of information, consistent with findings showing most health related information and particularly about health risk is obtained from the media [37,38]. It was common for participants to mention that their views on the pandemic and images of swine flu mainly came from the media. Participants assessed that in the early phase of the pandemic there was too much news coverage and associated this with deliberate 'scaremongering' to induce unnecessary worry. Whilst a systematic analysis of newsprint media coverage has found no evidence of exaggerating the content of reporting (Hilton in submission), there is evidence that participants found the high levels of swine flu coverage disconcerting. This resonates with the 'agenda setting' model which suggests that news topics which receive the most attention may be perceived to be the most important [39]. Participants also assessed that the media purposefully tried to induce worry which in turn increased their scepticism about the 'true' threat. This led some to state that they mistrusted the media as a reliable information source. Nevertheless, people also judged that exaggerative journalism tended to have a greater influence on others than on oneself.

## Conclusion

In conclusion, a criticism that has been levelled at the UK Government and the media is that it over-reacted to what turned out to be a mild pandemic. However, our findings show that there was little evidence of the public over-reacting or thinking the government had overreacted. Instead people believed contracting swine flu was inevitable and assessed self-efficacy for protecting against it to be low. This may lead to apathy about following recommended protective health behaviours. Importantly, people in this study assessed that a greater risk to their health was posed by the vaccine than the disease, which could have implications for the risk-benefit analysis people do when deciding about vaccination. Had the vaccination programme been rolled out there may have been sub-optimal vaccine uptake if people deemed the risk from swine flu as low and found it difficult to quantify risks connected to the safety of the vaccine. Further work needs to be done to explain seasonal influenza viruses, novel influenza strains and how influenza vaccines are developed each year to combat the threat posed to the public. We found that the government's public awareness campaign appeared to confirm what people already thought they knew but failed to challenge them to consider their own behaviour and therefore may have fallen short of encouraging people to adopt new health protective behaviours. Nevertheless, there was general support for the government's handling of the pandemic and for the media as a useful information source, which was deemed to have over-reported, rather than over-reacted, to the swine flu pandemic.

## Competing interests

The authors declare that they have no competing interests. This study was funded by the Medical Research Council.

## Authors' contributions

SH participated in the design, analysis, and drafted the manuscript. ES collected the data and commented on drafts of the manuscript. Both authors approved the final manuscript.

## Pre-publication history

The pre-publication history for this paper can be accessed here:

http://www.biomedcentral.com/1471-2458/10/697/prepub
